# Response to the letter regarding our article, ‘Potential predisposing features of external cervical resorption: An observational study’ (Patel et al., 2025)

**DOI:** 10.1111/iej.14216

**Published:** 2025-03-05

**Authors:** Shanon Patel, Francesc Abella, Kreena Patel, AbdulAziz Bakhsh, Paul Lambrechts, Nassr Al‐Nuaimi

**Affiliations:** ^1^ Faculty of Dentistry, Oral & Craniofacial Sciences King's College London UK; ^2^ Guy's & St Thomas NHS Foundation Trust London UK; ^3^ Private Practice London UK; ^4^ Universitat Internacional de Catalunya Barcelona Spain; ^5^ Department of Restorative Dentistry, Endodontic Division, Faculty of Dental Medicine Umm Al‐Qura University Makkah Saudi Arabia; ^6^ Department of Oral Health Sciences, Endodontology University Hospitals Leuven, KU Leuven Leuven Belgium; ^7^ Boston University Henry M. Goldman School of Dental Medicine Boston USA; ^8^ College of Dentistry University of Baghdad Baghdad Iraq

**Keywords:** external cervical resorption, predisposing factors

Dear Editor‐in‐Chief,

Thank you for providing the opportunity to respond to the letter regarding our article, *‘Potential Predisposing Features of External Cervical Resorption: An Observational Study’* (Patel et al., 2025). The thoughtful and constructive critique is appreciated and as authors we would like to address the concerns raised in the letter.

## USE OF THE TERM ‘PREDISPOSING FACTOR’

The concern regarding the use of the term ‘predisposing factor’ in the context of a cross‐sectional study is duly noted. We acknowledge the importance of precise language in scientific discourse. Our use of ‘predisposing factor’ aligns with existing literature and epidemiological conventions to describe characteristics associated with a condition without implying direct causation. Importantly, we explicitly mentioned in the title that our study is ‘an observational study’, indicating that no causality can be expected from the findings. As an observational study, we explicitly state in our discussion that causality cannot be inferred and that the identified factors are associations rather than definitive causes.

The inclusion of cat ownership as a potential factor was, of course, based on limited preliminary evidence, and while the mechanism we referenced is speculative, it was meant to present a hypothesis for future research rather than a definitive causal relationship. We are confident that the study's conclusions, as framed, are appropriately cautious regarding these associations.

## CONCERNS REGARDING STATISTICAL METHODS (CHI‐SQUARED TESTS)

The reviewer raised valid points about the limitations of chi‐squared tests in the context of complex, multivariable relationships, particularly with regard to confounding and clustering effects. Chi‐squared analysis was employed to assess associations between categorical variables, a widely accepted method in exploratory epidemiological research. While we acknowledge that multivariable regression models could further adjust for confounders, our study aimed primarily to identify potential associations rather than to determine adjusted risk estimates. Future studies employing regression modelling and causal inference frameworks are likely to provide deeper insights.

The hierarchical structure of our data (patients and teeth) introduces clustering effects, and we agree that more advanced methods could address this more effectively. While chi‐squared tests do not account for clustering, we would like to mention that 92.3% (179/194) of patients had only one tooth, which means there is no within‐patient clustering for these individuals. This substantially reduces the potential for clustering effects since these patients contribute only a single observation. Additionally, 12 (6.2%) patients had two teeth and only 3 (1.5%) patients had four teeth. While there is some within‐patient clustering for these patients (i.e. observations are not independent as the same patient contributes multiple data points), the proportion of patients with multiple teeth is small relative to the total number of patients. Therefore, the overall clustering effect is expected to be minimal.

## USE OF *p*‐VALUES AND STATISTICAL SIGNIFICANCE

There are valid concerns raised regarding the limitations of *p*‐values, which reflect broader discussions in the field of statistical methodology. In our study, *p*‐values were employed as a measure of statistical significance, and we fully acknowledge that *p*‐values, in isolation, do not capture the magnitude or clinical relevance of an effect. Our decision to report *p*‐values was based on their standard use in hypothesis testing, consistent with common practice in similar observational studies. However, we recognize that *p*‐values do not provide a comprehensive assessment of the strength or direction of associations.

In light of the sample size of 215 participants in our study, we note that the *p*‐value is less likely to be unduly influenced by sample size, as might be the case in studies with much larger sample sizes, which can detect minute differences that may not be of practical significance. Our sample size allows for a more meaningful and balanced interpretation of statistical significance, minimizing the risk of detecting trivial effects that could be magnified in studies with thousands of participants. Consequently, the *p*‐value in our study is reflective of the true effect, rather than being disproportionately affected by the sample size.

## MULTIPLE COMPARISONS AND FALSE DISCOVERY RATE

The concern regarding multiple comparisons and the potential for false positives is certainly a valid consideration in epidemiological research. In this study, we acknowledge that multiple statistical tests were conducted. However, the exploratory nature of the research was not intended to make definitive claims about the relationships between the variables, but rather to identify potential associations for further study. The study was designed with this in mind, and the results should be interpreted as suggestive rather than conclusive. We do not consider the *p*‐values in isolation but in the broader context of the existing literature on external cervical resorption.

## CONCLUSION

We believe that the methodology employed in our study was appropriate to address the research questions and that we used the correct study design. The use of chi‐squared tests, the reporting of *p*‐values and the exploration of potential associations were all consistent with accepted practices in observational research. Our study should be viewed as an initial investigation into the potential factors associated with external cervical resorption, and we stand by the rigor of the analysis presented.

We hope that our response clarifies the rationale behind our methodology and provides context for the decisions made. We appreciate the opportunity to address these concerns and are grateful for the continued interest in our work.

Thank you for your time and consideration.

Sincerely,

Shanon Patel.

## CONFLICT OF INTEREST STATEMENT

The authors deny any conflicts of interest.

## ETHICS APPROVAL

No ethical approval or informed consent is required.

## Data Availability

Data sharing is not applicable to this article as no new data were created or analyzed in this study.

